# Thoracic and Abdominal Complications After Posterior Fusion for Adolescent Idiopathic Scoliosis: Evidence and Clinical Implications

**DOI:** 10.3390/jcm15218573

**Published:** 2026-11-03

**Authors:** Michał Grabala, Piotr Kowalski, Justyna Walczak, Joanna Suszczyńska, Krzysztof Zakrzewski, Paweł Grabala

**Affiliations:** 1 2nd Clinical Department of General and Gastroenterological Surgery, Medical University of Bialystok, ul. M. Skłodowskiej-Curie 24A, 15-276 Bialystok, Poland; michal@grabala.pl (M.G.); joannasuszcz@gmail.com (J.S.); 2 Department of Neurosurgery, Regional Specialized Hospital, ul. Dekerta 1, 66-400 Gorzow, Poland; pkowal72@gmail.com; 3 Department of Neurosurgery, Polish Mother’s Memorial Hospital Research Institute, Rzgowska 281/289, 93-338 Lodz, Poland; justyna.walczak@iczmp.edu.pl (J.W.); krzysztof.zakrzewski@iczmp.edu.pl (K.Z.)

**Keywords:** scoliosis, adolescent, spinal fusion, postoperative complications, pleural effusion, ileus, perioperative care, adolescent idiopathic scoliosis, thoracic complications, abdominal complications, perioperative management

## Abstract

**Background:** Posterior spinal fusion (PSF) is an established treatment for progressive adolescent idiopathic scoliosis (AIS) requiring surgery. Thoracic and abdominal complications range from transient postoperative findings to uncommon emergencies. This narrative review examines their reported frequency, recognition, prevention, and management. **Methods:** Literature published between January 2000 and June 2026 was reviewed using PubMed/MEDLINE and Google Scholar, supplemented by reference-list screening. AIS-specific evidence was prioritized. Reports involving other deformities or surgical approaches and general management guidelines were used as explicitly identified contextual evidence. No pooled incidence estimates were calculated. **Results:** Reported frequencies depended strongly on outcome definitions, imaging protocols, surgical approach, and follow-up. Two retrospective AIS studies detected pleural effusion in 71.0% and 76.3% of patients on computed tomography approximately one week after PSF; in the latter study, all effusions were asymptomatic and required no treatment. These findings should not be equated with clinically significant pulmonary complications. Postoperative ileus was reported in 13.2% of one 318-patient AIS cohort aged 10–20 years, whereas superior mesenteric artery syndrome, chylothorax, hemothorax, and implant-related vascular injury were supported largely by small series or case reports. Enhanced recovery pathways were consistently associated with shorter hospitalization, but evidence that they prevent individual rare complications was limited. Accurate instrumentation, appropriate blood conservation, individualized nutritional assessment, opioid-sparing analgesia, and symptom-directed investigation support perioperative care. **Conclusions:** Thoracic and abdominal complications after PSF for AIS require separate interpretation of radiographic findings, clinically diagnosed events, and events requiring intervention. Persistent vomiting, progressive abdominal distension, respiratory deterioration, unexplained anemia, or hemodynamic instability warrant prompt reassessment. Management should follow the suspected diagnosis, patient physiology, and relevant pediatric or multidisciplinary guidance.

## 1. Introduction

Adolescent idiopathic scoliosis (AIS) is a three-dimensional spinal deformity of unknown cause that develops after ten years of age. Estimates of prevalence vary with the population and diagnostic threshold; a contemporary review reports that AIS affects approximately 2–4% of adolescents. Severe curves occur more frequently in girls. Decisions about surgery depend on curve magnitude, remaining growth, progression, symptoms, and patient preferences. Posterior spinal fusion (PSF) is an established option for progressive deformity requiring operative correction [1].

A recent database publication documents a substantial annual volume of posterior fusion for AIS, although the underlying administrative data precede publication. In the U.S. National Readmissions Database analysis reported by Singh et al., the annual cohorts contained 7088 posterior fusion admissions coded for AIS in 2016 and 7545 in 2019 [2]. The analysis included operations from January through November of each year to permit 30-day follow-up and excluded robotic procedures. These are counts within the reported database analysis, rather than a complete calendar-year census or an estimate of current worldwide utilization; diagnosis-code identification also does not guarantee a strictly adolescent age distribution. Nevertheless, the number of admissions demonstrates why preventing avoidable morbidity and recognizing uncommon serious events matters at the population level.

**Figure 1:**
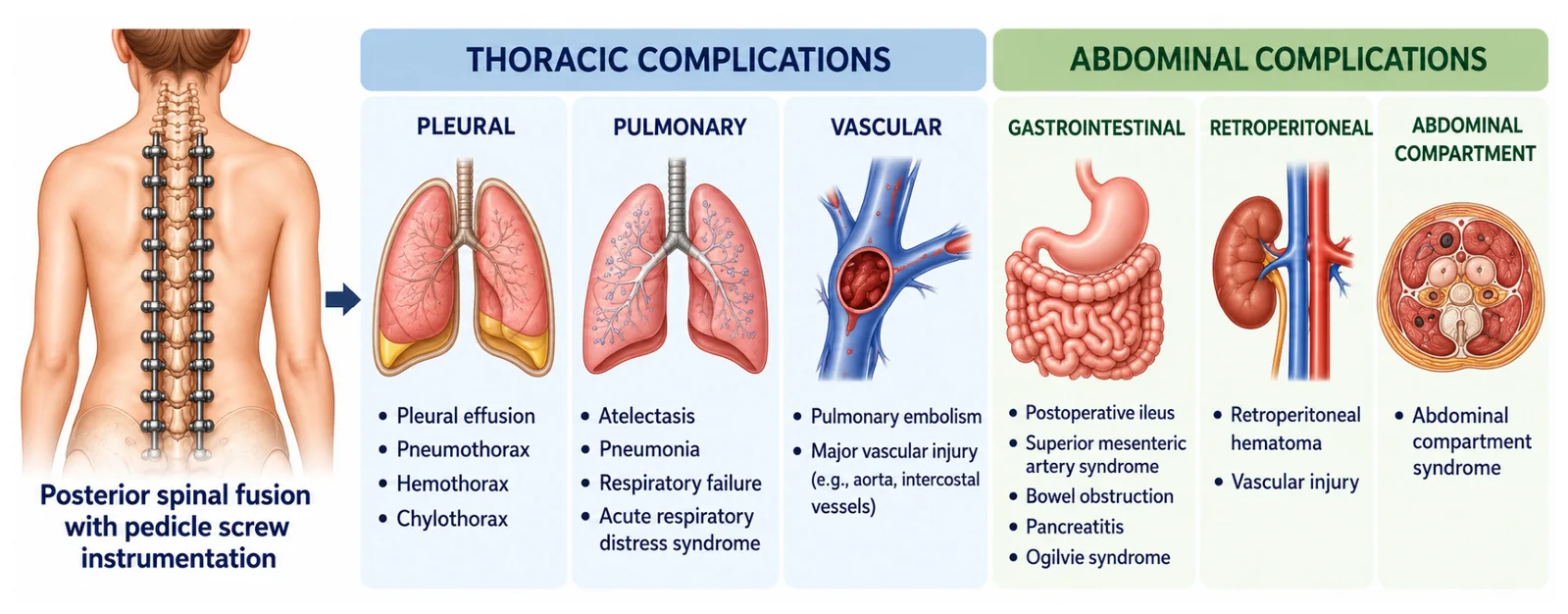
Overview of thoracic and abdominal complications after posterior spinal fusion for adolescent idiopathic scoliosis. Although most complications are uncommon, delayed diagnosis may lead to significant morbidity, prolonged hospitalization, and increased health care costs. Awareness, early detection, and prompt management are essential to optimize outcomes in adolescents undergoing PSF. The figure was prepared using code developed with assistance from ChatGPT (OpenAI, USA; https://chatgpt.com/; accessed on 17 September 2026). The authors take responsibility for the figure’s content and accuracy.

The physiological burden of PSF includes tissue dissection, blood loss, prolonged anesthesia, altered postoperative mobility, and exposure to opioid analgesics. However, complication rates cannot be described meaningfully without their definitions and observation periods. In a registry study of 3582 surgically treated patients with AIS, Bartley et al. reported a 2.6% rate of major perioperative complications across different approaches, including pulmonary and gastrointestinal events in 0.4% and 0.2%, respectively. The major perioperative complication rate was 2.4% in the posterior-only subgroup. These figures describe selected major events and should not be applied to all postoperative symptoms or imaging abnormalities [3]. Figure 1 summarizes thoracic and abdominal complications after posterior spinal fusion for adolescent idiopathic scoliosis.

In another cohort of 1057 patients undergoing PSF for AIS, Kwan et al. reported major and minor complication rates of 0.95% and 1.32%, respectively; the age range was 10–30 years, which limits direct application to a strictly adolescent population [4]. Keil et al., using a broader definition in 424 adolescents, reported complications in 47% when persistent pain was included and in 24% when persistent pain was excluded [5]. These estimates are not contradictory: they measure different outcomes in different settings.

This review organizes the evidence on thoracic and abdominal complications after PSF for AIS into a clinically useful approach. It distinguishes routine recovery from evolving respiratory, vascular, or abdominal emergencies and interprets event frequencies according to their definitions, populations, and observation periods.

## 2. Materials and Methods

### 2.1. Review Design and Literature Search

This narrative review examined the reported frequency, clinical presentation, prevention, diagnosis, and management of thoracic and abdominal complications after posterior spinal fusion (PSF) for adolescent idiopathic scoliosis (AIS). The literature search used PubMed/MEDLINE and Google Scholar and covered publications from 1 January 2000 to 30 June 2026. Articles available online ahead of print within this period were eligible for consideration.

Searches combined terms describing the underlying condition and procedure, including adolescent idiopathic scoliosis, posterior spinal fusion, and posterior spinal instrumentation, with terms describing thoracic and gastrointestinal complications. Complication-specific terms included pleural effusion, pneumothorax, hemothorax, chylothorax, postoperative ileus, superior mesenteric artery syndrome, bowel obstruction, Ogilvie syndrome, vascular injury, aortic injury, retroperitoneal hematoma, and psoas hematoma. Medical Subject Headings and free-text terms were used in PubMed/MEDLINE, and keyword searches were used in Google Scholar. Reference lists of relevant publications were also screened to identify additional sources.

English-language publications formed the main evidence base. A relevant Czech-language case report with an English abstract was additionally included as contextual evidence concerning vascular compression and pancreatitis after scoliosis correction. Its inclusion constituted an exception to the language preference.

PubMed/MEDLINE was the principal indexed biomedical source. Google Scholar was used as a supplementary academic search engine to identify additional relevant publications alongside the reference-list screening described above, which provided backward citation tracking. It was not treated as an interchangeable primary bibliographic database. Its broader coverage can help locate relevant publications, but opaque ranking, variable retrieval, and limited reproducibility restrict its use as a stand-alone review source [6]. The evidence presented here comprises published journal articles and professional guidance; no separate gray-literature evidence base or systematic gray-literature search is claimed.

### 2.2. Scope and Selection of Evidence

Clinical relevance, study design, and applicability to PSF for AIS guided source selection. Eligible publication types included randomized trials, prospective and retrospective cohort studies, registry and administrative database analyses, systematic reviews, narrative reviews, case series, case reports, and clinical practice guidelines. Comparative studies of perioperative interventions and enhanced recovery pathways were included when they addressed outcomes relevant to complication prevention or postoperative recovery.

Studies of PSF for AIS formed the primary clinical evidence base. Where cohorts included young adults alongside adolescents, the reported age range was considered when interpreting applicability. Studies involving juvenile idiopathic, congenital, neuromuscular, syndromic, or adult spinal deformities, as well as mixed surgical approaches, were retained as contextual evidence when they provided relevant information about rare complications, anatomical mechanisms, or management. Their findings were not presented as complication rates specific to posterior-only AIS surgery unless an appropriate subgroup was reported separately.

Case reports and small series were used to describe unusual presentations, delayed events, and potential management responses. They were not used to estimate population incidence or comparative treatment effectiveness. Pediatric guidance was prioritized for diagnostic and management principles. Adult and general surgical guidance was identified as extrapolated evidence where directly applicable pediatric or AIS-specific evidence was limited. Publications without a relevant contribution to the clinical scope of the review were excluded.

**Figure 2:**
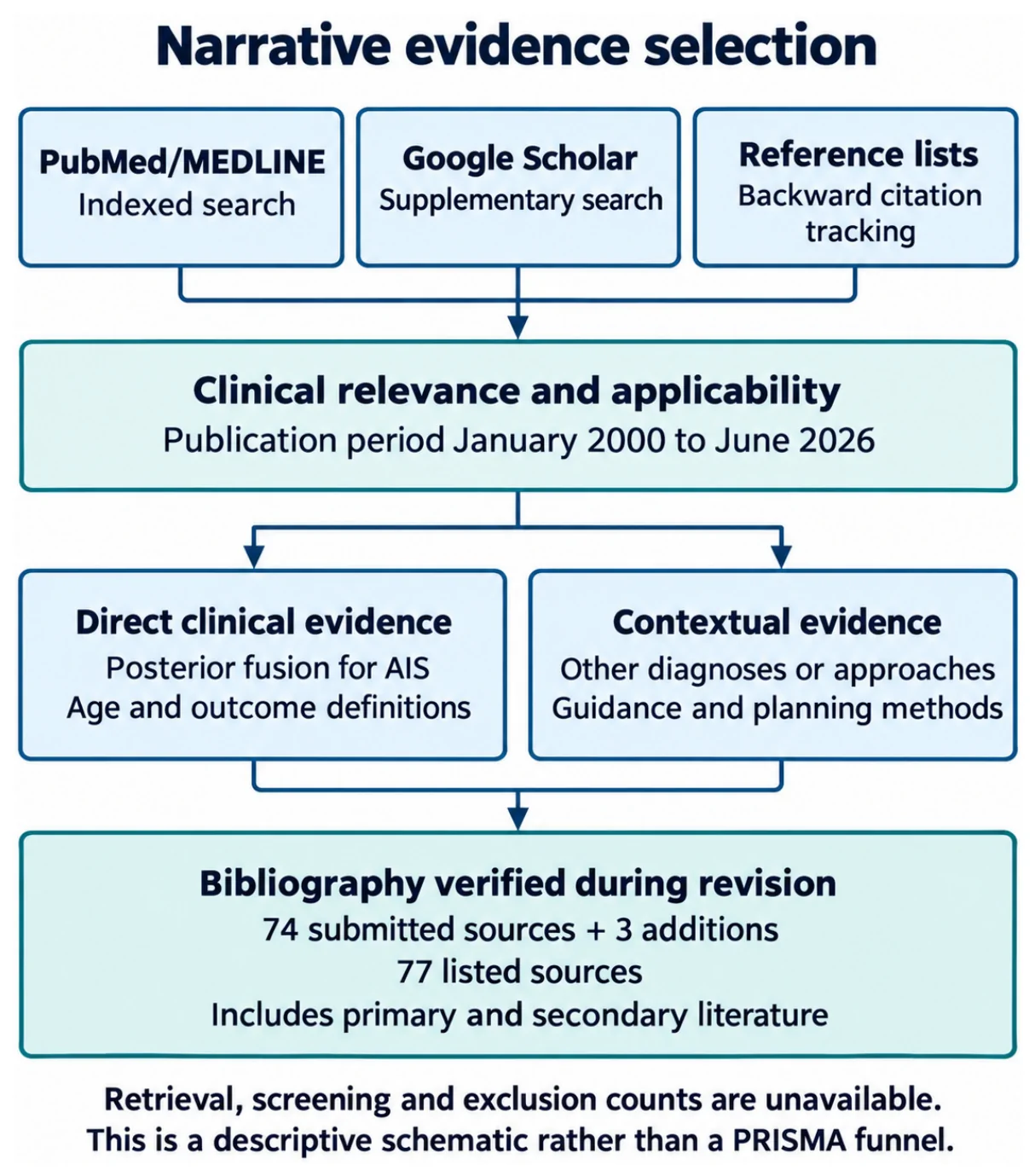
Source-selection schematic for the narrative review. Only the bibliography counts can be verified from the available review documentation. The diagram does not imply prospectively recorded retrieval, screening, or exclusion counts, and the 77 sources are not all primary studies. AIS, adolescent idiopathic scoliosis; PRISMA, Preferred Reporting Items for Systematic Reviews and Meta-Analyses.

The submitted bibliography contained 74 publications. Three publications were added during revision, producing a final bibliography of 77 sources, comprising primary research, evidence syntheses, case reports, and professional guidance. This is a count of listed publications, not 77 independent primary studies or a count of all full texts assessed. Exact numbers of records initially retrieved, screened, and excluded were not available in the documentation used for this revision and cannot be reliably re-constructed from the bibliography. Consequently, no numerical screening funnel or database-specific retrieval totals are asserted. Figure 2 shows the source-selection approach and the verifiable bibliography counts; it is a descriptive schematic, not a PRISMA flow diagram. Incomplete screening documentation limits reproducibility and leaves the possibility of selection bias.

### 2.3. Data Extraction and Narrative Synthesis

Information extracted from the selected publications included study design, sample size, age range, scoliosis diagnosis, surgical approach, complication definition, method of detection, follow-up period, event counts or reported frequencies, associated factors, interventions, and clinical outcomes. Interpretation distinguished patient-level outcomes from screw-level measurements and separated radiographic abnormalities from symptomatic complications and events requiring treatment.

Findings were organized by complication, with additional synthesis addressing prevention, perioperative care, and enhanced recovery. Differences between studies were interpreted in relation to case mix, surveillance intensity, diagnostic criteria, operative practice, and follow-up. Observational associations were not treated as evidence of causality. A nonsignificant difference was not interpreted as proof of equivalence or absence of risk, and shorter hospitalization was distinguished from demonstrated prevention of specific complications.

Study limitations and clinical applicability were considered qualitatively. No pooled incidence estimates, meta-analysis, or formal certainty grading were undertaken. Practical implications were formulated by integrating directly applicable evidence with contextual reports and relevant guidance, while acknowledging uncertainty where conclusions depended on small observational studies, case reports, or extrapolation.

## 3. Thoracic Complications

### 3.1. Pleural Effusion

Pleural fluid is frequently detected when sensitive imaging is performed after PSF, but imaging prevalence differs from the frequency of clinically important pleural disease. Hayashi et al. retrospectively analyzed prospectively collected data from 76 patients with AIS and identified pleural fluid in 54/76 patients (71.0%) on computed tomography (CT) at one week. Fluid was bilateral in 36 patients, corresponding to 47.4% of the entire cohort or 66.7% of patients with effusion. Fusion of at least 11 levels was associated with effusion (odds ratio [OR], 3.88; 95% confidence interval [CI], 1.31–11.47) [7]. In a matched cohort study of 54 patients undergoing spinal deformity correction, O-arm navigation was associated with a lower mean postoperative pleural effusion volume (*p* < 0.001) and a lower screw perforation rate (*p* = 0.007) than the free-hand technique. In both groups, patients with malpositioned screws had significantly greater effusion volumes than those without screw malposition [8]. Ozaki et al. retrospectively reviewed 114 patients and found pleural effusion in 87/114 (76.3%) at one week. All affected patients were asymptomatic and required no additional treatment. Pulmonary function and lung-volume measurements at two years did not differ significantly between patients with and without early effusion [9]. Thoracoplasty changes the clinical context. In a retrospective series of 45 patients undergoing PSF with concomitant thoracoplasty, Amanullah et al. reported effusion in 16/45 (35.6%) and chest-tube placement in 3/45 (6.7%). Deep subfascial drains did not significantly reduce effusion occurrence [10].

The natural history of incidental postoperative fluid appears favorable in the cohort of Ozaki et al.: no residual effusion was present at the two-year assessment despite the absence of effusion-specific treatment, supporting spontaneous resolution in that setting [9]. However, CT assessment at one week and two years does not establish whether resolution occurred over days, weeks, or months. No progression to symptomatic effusion was reported in this cohort, but its retrospective design and limited sample do not support a reliable population estimate of progression risk or a conclusion that the risk is zero. The two-year pulmonary findings also do not establish an identical early recovery trajectory in every patient.

Evaluation should follow symptoms, oxygen requirement, examination, and the operative history. Ultrasound or chest radiography can assess suspected pleural fluid; CT is reserved for unresolved diagnostic questions or suspected associated injury. Small asymptomatic effusions can usually be observed. Respiratory compromise, progressive accumulation, suspected infection, chyle, or bleeding requires investigation and consideration of drainage. Drainage decisions should account for the suspected cause, fluid accumulation, and physiological effect.

Accordingly, an incidental small collection in a clinically stable adolescent can be followed by clinical reassessment, without drainage or repeated CT solely to document disappearance. Increasing dyspnea, fever, pleuritic pain, a falling hemoglobin concentration, or a rising oxygen requirement changes the assessment and warrants targeted imaging and evaluation of the cause. The higher intervention burden in patients undergoing thoracoplasty should be considered separately [10]. A new or enlarging collection should not automatically be labeled the same benign finding as an asymptomatic early CT effusion.

### 3.2. Pneumothorax

Pleural injury may occur during instrumentation or thoracoplasty. Esposito et al. reported pneumothorax in 30/9036 admissions (0.3%) in the Kids’ Inpatient Database, with intervention in 4/9036 (0.04%). Their institutional series identified pneumothorax in 8/244 patients (3.3%), none requiring intervention [11]. Differences in ascertainment help explain the variation between datasets.

New hypoxemia, pleuritic pain, asymmetric breath sounds, or unexplained ventilatory difficulty should prompt assessment. A stable patient can undergo chest radiography or bedside ultrasound. Suspected tension pneumothorax with physiological compromise requires immediate decompression without waiting for confirmatory imaging. Other pneumothoraces are managed according to symptoms, physiological stability, progression, and the need for positive-pressure ventilation. These emergency principles are extrapolated from general thoracic injury guidance rather than AIS comparative trials [12]. The findings of Esposito et al. support selective postoperative chest radiography guided by clinical and operative concerns [11].

### 3.3. Chylothorax

Chylothorax after posterior scoliosis surgery is described mainly in individual reports. A reliable AIS-specific incidence cannot be calculated from these reports. Van-Loo et al. described a 16-year-old girl with chylothorax on postoperative day three after PSF, treated with pleural drainage and dietary restriction of long-chain fat [13]. Weening et al. reported chylothorax and chyluria after posterior instrumentation and discussed thoracic duct obstruction as a possible mechanism [14].

Pleural fluid may be milky, but fasting or poor nutritional intake can alter its appearance. General chylothorax literature supports measurement of pleural triglycerides and cholesterol, with chylomicron testing when the biochemical result is equivocal. Triglycerides above 110 mg/dL with cholesterol below 200 mg/dL support the diagnosis; demonstration of chylomicrons is confirmatory. These criteria derive from broader chylothorax populations [15].

Treatment requires attention to respiratory status, drainage losses, electrolytes, protein, and nutrition. A diet low in long-chain triglycerides with medium-chain triglyceride supplementation may be appropriate; selected patients require temporary parenteral nutrition. Octreotide, lymphatic imaging, embolization, or surgery may be considered when conservative treatment is unsuccessful. Escalation should be individualized to the adolescent patient. Decisions depend on body size, output trajectory, nutritional consequences, and specialist assessment [15].

### 3.4. Pneumonia, Atelectasis, and Respiratory Failure

Basques et al. identified pneumonia in 1/733 patients (0.1%) undergoing PSF for AIS in a pediatric surgical database with 30-day follow-up [16]. A radiographic study by Hussein and Black identified atelectasis in 50/167 pediatric patients (29.9%) within seven postoperative days. That cohort included different scoliosis diagnoses and was evaluated through postoperative chest imaging, so its result describes radiographic abnormalities in a broader pediatric population [17]. Pain-limited breathing, retained secretions, residual sedation, aspiration, and pre-existing respiratory disease should be considered when respiratory recovery is delayed. Assessment includes oxygenation, ventilation, examination, and targeted imaging. A radiographic opacity alone does not distinguish atelectasis from pneumonia. Analgesia that permits effective breathing, mobilization when stable, and respiratory physiotherapy are reasonable components of care. Antibiotic treatment should follow a clinical diagnosis of infection and relevant local guidance rather than isolated postoperative radiographic changes.

Extubation and postoperative monitoring should be individualized to respiratory reserve and intraoperative events. Hypoxemia requires evaluation for reversible causes, including mucus plugging, effusion, pneumothorax, aspiration, bleeding, and thromboembolism. Evidence on enhanced recovery after surgery (ERAS) pathways primarily concerns hospital stay and recovery milestones, with less information on respiratory failure [18]. Pulmonary events in mixed-approach AIS registries provide additional context [3].

### 3.5. Hemothorax

Hemothorax can follow pleural or vascular injury and may present with respiratory deterioration, chest pain, a falling hemoglobin concentration, or shock. Direct AIS evidence consists largely of case reports rather than denominator-based estimates [19,20]. A 2026 report described delayed hemothorax five years after AIS fusion, with a thoracic screw in contact with the aorta on CT angiography [21]. Delayed presentation should therefore remain in the differential diagnosis when new thoracic symptoms develop in an instrumented patient.

Resuscitation, pleural assessment, and early thoracic or vascular consultation are priorities. CT angiography is useful when the patient is sufficiently stable and vascular injury is suspected. Drainage, operative exploration, or endovascular treatment depends on the bleeding source and physiology, consistent with general thoracic injury guidance [12].

### 3.6. Venous Thromboembolism

Rudic et al. identified 38 clinically recorded venous thromboembolism (VTE) events among 11,775 patients (0.323%) within 90 days of instrumented fusion for AIS. Associated factors included thrombophilia, longer fusion, obesity, and older adolescent age [22]. In a retrospective study of 1471 pediatric patients undergoing 2131 posterior spinal fusion procedures without pharmacological thromboprophylaxis or routine VTE screening, symptomatic deep vein thrombosis (DVT) occurred in two patients (2/1471), both with neuromuscular scoliosis, 55–161 days after surgery. Neither patient developed pulmonary embolism [23]. A systematic review of pediatric orthopaedic populations aged up to 21 years estimated a pooled VTE incidence of 16.6 per 10,000 patients (95% CI, 9.1–30.5) across 26 studies involving 850,268 patients, after exclusions that included osteomyelitis cohorts. Higher rates were observed in trauma cohorts. Frequently investigated potential risk factors included age, sex, obesity, surgery type, and central venous catheter use. These findings provide broader orthopaedic context and are not specific to AIS fusion [24].

Early mobilization and individualized assessment of thrombosis and bleeding risk are appropriate. Mechanical prophylaxis should follow pediatric institutional practice and device suitability. The 2026 guideline from the American Society of Hematology and the International Society on Thrombosis and Haemostasis conditionally suggests against routine anticoagulant prophylaxis for pediatric noncardiac surgery, based on very low-certainty evidence, while emphasizing individual risk assessment. This recommendation covers pediatric noncardiac surgery broadly; mechanical methods and aspirin were outside the guideline scope [25].

Unexplained tachycardia, hypoxemia, pleuritic pain, syncope, or unilateral limb swelling requires prompt assessment. Diagnostic testing should be selected for the clinical presentation, including venous ultrasound or pulmonary vascular imaging when indicated. Postoperative D-dimer elevation is nonspecific and should not independently establish the diagnosis. Confirmed VTE requires coordination among the spine team, pediatric hematology, and critical care as appropriate, taking recent surgery and the consequences of bleeding into account.

### 3.7. Implant-Related Vascular and Visceral Injury

A CT-defined screw breach is not equivalent to clinical injury. Abul-Kasim et al. evaluated 873 screws in 49 patients with AIS and classified 149/873 screws (17%) as misplaced, without observed neurovascular complications [26]. In a multicenter cohort of 318 children with mixed spinal deformities and postoperative CT assessment, 98% of 6358 free-hand pedicle screws were classified as acceptably positioned, including grade 0, grade 1, and juxtapedicular placements. Breaches were reported in 5.92% of screws; 0.88% breached more than 4 mm, and 0.26% were replaced. No new permanent neurological, vascular, or visceral complications were reported. These percentages use screws as the denominator and depend on the classification and selection of patients undergoing CT [27]. Structures at risk vary with vertebral level, rotation, and screw trajectory [28]. In a retrospective cohort of 230 children aged 2–18 years with mixed spinal deformities, 5080 pedicle screws were assessed by comparing preoperative magnetic resonance imaging (MRI) with postoperative CT. Reported mean increases in pedicle diameter ranged from 1.90 to 2.92 mm (47–71%) across T1–L5, without breach-related complications requiring revision. The different imaging modalities limit attribution of the entire measured change to mechanical expansion, and these observations do not establish a universal rule for screw oversizing [29].

Delayed visceral injury is illustrated by an AIS case in which a malpositioned T4 pedicle screw caused esophageal perforation and dysphagia 10 years after posterior instrumented fusion. Implant removal and nasogastric tube placement were followed by complete healing within 12 weeks without complications [30]. Delayed aortic contact or penetration requires careful planning even when symptoms are absent. Contextual reports include a patient with Prader–Willi syndrome and an older adult with degenerative scoliosis [31,32]. A screw suspected of penetrating or closely involving a major vessel should not be removed before vascular imaging and a plan for vascular control. Open repair, endovascular treatment, or surveillance is selected case by case by the relevant specialists.

Accurate preoperative planning, appropriate screw length and trajectory, and intraoperative assessment remain central. Somatosensory and motor evoked potentials assess neural function; normal neuromonitoring does not exclude pleural or vascular injury. The uncontrolled O-arm series by Chang et al. provides descriptive AIS experience without a comparison group [33]. Comparative observational results differ: a pediatric idiopathic scoliosis database study associated navigation with fewer neurological injuries and transfusions [34], whereas an AIS study associated computer-assisted navigation with higher recorded complication and transfusion rates [2]. Differences in case complexity, coding, and selection limit causal interpretation. Table 1 summarizes selected thoracic outcomes and their clinical interpretation.

## 4. Abdominal and Gastrointestinal Complications

Abdominal complications can develop after a posterior operation even when the peritoneal cavity is not entered. Gastrointestinal recovery reflects the combined effects of perioperative medication, physiological stress, mobility, nutritional reserve, and the anatomical change produced by deformity correction. Distinguishing impaired motility from mechanical obstruction or visceral ischemia is essential because similar early symptoms can require different treatment [35,36,37,38]. Opioid-related bowel dysfunction is one potentially modifiable contributor. Opioids suppress propulsive intestinal motility and can produce constipation, nausea, and delayed gastric or intestinal transit. Anesthesia and the surgical stress response may further impair motility, while pain-limited movement, reduced intake, dehydration, and electrolyte disturbance can prolong gastrointestinal recovery. These mechanisms provide a rationale for maintaining effective multimodal analgesia while limiting unnecessary opioid exposure, correcting fluid and electrolyte deficits, and restoring oral intake and mobility as tolerated. AIS-specific comparative evidence for individual preventive components remains limited, and the relative contribution of each mechanism cannot be quantified from the available studies [35,36,39].

Correction can also change the relationship between the spine, mesentery, and abdominal contents. Axial lengthening and altered aortomesenteric geometry may contribute to duodenal compression in a susceptible patient, particularly when a limited mesenteric fat cushion is compounded by poor intake or postoperative weight loss [37,40,41]. This mechanical explanation for SMAS differs from the much rarer vascular-compression mechanism proposed in reports of visceral ischemia and pancreatitis [38]. Hypoperfusion, concealed bleeding, retroperitoneal irritation from an anteriorly breached screw, and external abdominal compression during positioning are additional considerations in an abnormal recovery trajectory [26,35,42,43,44]. These associations and case observations do not establish a correction threshold that prevents abdominal complications. Persistent bilious vomiting, focal pain, progressive distension, or systemic deterioration therefore requires diagnosis-specific reassessment rather than attribution to opioids alone.

### 4.1. Postoperative Ileus and Constipation

Transient delay in bowel function is common after major surgery, but constipation and postoperative ileus (POI) are not interchangeable. Zhang et al. reported POI in 42/318 patients with AIS aged 10–20 years (13.2%). Their definition required at least two specified gastrointestinal features at 72 h postoperatively, including nausea or vomiting, intolerance of oral intake, absent flatus, distension, or radiographic evidence [35]. Comparisons with other cohorts require comparable definitions and follow-up. In that study, larger changes in thoracolumbar and lumbar height and anterior lumbar screw breach were associated with POI. These retrospective associations require prospective confirmation before they can guide a preventive correction target or screw strategy [35].

Clinical management begins with reassessment of medication exposure, hydration, electrolytes, abdominal findings, and the pattern of recovery. Opioid exposure, immobility, and poor intake should be considered. Opioid-sparing analgesia and mobilization can support recovery, and oral intake should advance as tolerated when obstruction is not suspected. Persistent bilious vomiting, focal or worsening pain, marked distension, peritoneal signs, or systemic deterioration requires investigation for another diagnosis. Nasogastric decompression may be needed for substantial vomiting or distension. These measures represent a clinical approach to postoperative recovery; evidence comparing specific AIS bowel regimens remains limited [36]. A systematic review of six studies comprising 468 patients found limited evidence that any particular bowel regimen reduces postoperative bowel morbidity or hospitalization [36]. In a matched retrospective study of 52 treated patients and 52 controls, oral methylnaltrexone was associated with more bowel movements by postoperative day two (31/52 versus 16/52), less abdominal distension (17% versus 40%), and shorter stay (3.09 versus 3.69 days) [39]. These results do not establish routine pediatric use or prevention of formally defined POI; any use requires specialist consideration of pediatric prescribing status and contraindications, particularly suspected mechanical obstruction. A 60-patient randomized trial found no significant benefit of chewing gum for bowel recovery or length of stay [45]. A meta-analysis of three randomized trials likewise found no significant improvement in postoperative abdominal pain, nausea, or hospital stay after PSF for AIS [46]. In another randomized trial involving 83 patients undergoing PSF for AIS, postoperative gum chewing was associated with an earlier first bowel movement (mean difference, 30.9 h; *p* = 0.04). No significant differences were observed in time to first flatus, length of hospital stay, or subjective gastrointestinal symptoms [47].

### 4.2. Superior Mesenteric Artery Syndrome

Superior mesenteric artery syndrome (SMAS) involves compression of the third duodenal portion between the aorta and superior mesenteric artery. Low nutritional reserve, postoperative weight loss, and anatomical changes after deformity correction may contribute [37,48]. Kwan et al. recorded two cases among 1057 patients (0.19%) in their AIS cohort, which included young adults [4]. Pappalardo et al. reviewed 61 reported patients from 29 publications involving different surgical approaches. This case-based synthesis describes presentation and treatment but does not provide a pooled incidence after posterior-only AIS surgery [37].

Symptoms include postprandial discomfort, early satiety, vomiting, abdominal distension, and inability to maintain intake. Bilious vomiting or failure to recover after an initial improvement should raise concern for obstruction. The review by Pappalardo et al. reported a mean onset of 69 days, with a range from three days to four years, supporting attention to symptoms after discharge [37]. This wide range describes selected published cases rather than the expected onset in an unselected AIS population.

Diagnosis requires a compatible clinical syndrome and evidence of duodenal obstruction. Upper gastrointestinal contrast examination or cross-sectional imaging can identify the obstruction and evaluate alternative causes. A reduced aortomesenteric angle or distance supports the diagnosis but does not establish symptomatic SMAS by itself [37,48].

Management principles combine reports after scoliosis surgery with general SMAS guidance [37,48,49]. Initial treatment includes correction of fluid and electrolyte deficits, gastric decompression when indicated, and nutritional rehabilitation. Enteral feeding distal to the obstruction is preferred when feasible; parenteral support may be necessary. Dietetic involvement and monitoring of weight and intake are essential. Persistent obstruction or failure to restore nutrition warrants surgical review. Duodenojejunostomy is an option for refractory disease, with timing determined by clinical response and nutritional progress [37,49].

### 4.3. Acute Pancreatitis

The frequency of acute pancreatitis after contemporary posterior-only AIS surgery remains uncertain. In a Nationwide Inpatient Sample analysis of 36,335 adolescents aged 10–18 years undergoing spinal fusion for idiopathic scoliosis between 2002 and 2011, pancreatitis was reported in ≤0.2% during the index hospitalization. This was an administrative analysis that included posterior, anterior, and combined approaches [50]. Laplaza et al. retrospectively reviewed 80 patients operated on in 1995–1997; 12 underwent enzyme testing for abdominal symptoms, and seven (approximately 9% of the full cohort) had enzyme elevations described as consistent with pancreatitis. The historical diagnostic criteria and selective testing limit comparison with contemporary pediatric diagnoses [51]. Feng et al. prospectively studied 176 patients undergoing posterior correction of scoliosis and diagnosed acute pancreatitis in 13 (7.4%) using clinical and laboratory findings [42]. This result is presented separately as contextual evidence from posterior scoliosis surgery, rather than as an AIS-specific incidence estimate. Differences in populations, diagnostic definitions, and ascertainment preclude pooling these figures. El Bouyousfi et al. described 14 adolescents with postoperative pancreatitis, with onset 4–51 days after scoliosis surgery and abdominal pain reported in only half of the cases [52]. Včelák et al. described two patients with idiopathic scoliosis, one with juvenile-onset disease and one with AIS, who developed postoperative pancreatitis attributed to vascular compression after posterior correction and fusion [38]. This mechanism should be considered when early abdominal deterioration suggests a vascular complication. The clinical report from the North American Society for Pediatric Gastroenterology, Hepatology and Nutrition recommends diagnosing pediatric acute pancreatitis when at least two of three criteria are present: compatible abdominal pain, serum amylase or lipase at least three times the upper limit of normal, and characteristic imaging findings. An isolated enzyme elevation does not suffice, and CT is not mandatory when the diagnosis is otherwise established [53].

Persistent vomiting, epigastric pain, or unexplained delayed gastrointestinal recovery should prompt consideration of pancreatitis and competing diagnoses. Management includes analgesia, early oral or enteral nutrition when tolerated, and individualized fluid administration with frequent reassessment of perfusion, urine output, renal function, respiratory status, and signs of fluid overload [53].

### 4.4. Acute Colonic Pseudo-Obstruction

Acute colonic pseudo-obstruction (ACPO, Ogilvie syndrome) is marked colonic dilatation without a mechanical obstructing lesion. Its incidence after AIS PSF is not established. Tsirikos and Sud described a 12-year-old with Chiari malformation and syringomyelia whose postoperative course progressed to perforation requiring right hemicolectomy [54]. This contextual report illustrates the consequences of delayed recognition in pediatric deformity surgery.

Progressive distension requires clinical assessment and imaging to exclude mechanical obstruction and identify ischemia or perforation. Initial measures include bowel rest when appropriate, correction of precipitating factors, reduction in offending medication, and decompression where indicated. Adult colorectal guidance supports monitored neostigmine or endoscopic decompression in selected patients who do not improve [55]. This is extrapolated guidance: adult drug dosing and adult diameter thresholds should not be adopted automatically for children. Pediatric surgical and gastroenterology input is appropriate. Peritoneal signs, ischemia, or perforation require urgent operative assessment.

### 4.5. Retroperitoneal or Psoas Hematoma and Visceral Injury

Unexplained anemia, flank or abdominal pain, femoral neuropathic symptoms, or hemodynamic deterioration should prompt consideration of concealed bleeding. Anatomical concern is particularly relevant to anterior or lateral vertebral breaches and adjacent segmental or major vessels. CT angiography may be required to identify active bleeding and its relation to instrumentation. The screw-placement literature informs anatomical risk but does not provide a reliable AIS-specific rate of retroperitoneal hematoma [26].

Management depends on physiological stability, ongoing blood loss, and the responsible lesion. Observation may be appropriate for a stable, nonexpanding hematoma after the cause has been assessed. Active arterial bleeding may require embolization or surgery. Drainage alone is not treatment for uncontrolled arterial hemorrhage, and revision of a screw adjacent to a major vessel requires a coordinated vascular plan. These principles are clinical extrapolations; comparative AIS treatment studies are lacking.

### 4.6. Abdominal Compartment Syndrome

Abdominal compartment syndrome (ACS) is a physiological emergency involving elevated intra-abdominal pressure and new or worsening organ dysfunction. The pediatric consensus definition uses sustained intra-abdominal pressure above 10 mmHg with attributable organ dysfunction [43]. Abdominal distension alone does not establish ACS.

When suspected, assessment includes hemodynamics, ventilation, urine output, abdominal examination, and pressure measurement using an age-appropriate standardized technique. Management targets the cause, intraluminal distension, fluid balance, and abdominal wall compliance. Overt ACS that does not respond to appropriate measures requires urgent surgical evaluation for decompression [43]. A reliable incidence after AIS PSF has not been established. Positioning that avoids unnecessary abdominal compression and timely recognition of evolving organ dysfunction are sensible preventive measures, but their specific effect on AIS-associated ACS has not been quantified.

### 4.7. Other Causes of Postoperative Abdominal Pain

New abdominal disease can coincide with recovery from spinal surgery. Aya et al. described appendicitis in a 14-year-old after AIS fusion [56]. Focal pain, fever, peritoneal findings, or persistent deterioration should prompt investigation beyond routine postoperative bowel dysfunction. Pediatric imaging should be selected according to clinical risk; ultrasound is often the initial examination for suspected appendicitis, with MRI or CT used when appropriate [57]. Table 2 summarizes abdominal outcomes, populations, and the limits of available frequency estimates.

## 5. Prevention and Perioperative Care

### 5.1. Preoperative Optimization

Preoperative assessment should identify recent weight loss, poor intake, gastrointestinal symptoms, anemia, respiratory disease, and personal or family thrombotic history. Nutritional assessment in adolescents should use age- and sex-appropriate growth measures and longitudinal weight change. Reports linking underweight status with SMAS support attention to nutritional vulnerability, although a universal preoperative weight-gain target or proven prevention regimen has not been established [40,41].

Dietetic referral is appropriate when intake or nutritional reserve is inadequate. Documented deficiencies should be managed for their established clinical indications. In an AIS cohort aged 10.6–25.3 years, Beling et al. found no difference in pain trajectories during the first 72 postoperative hours across vitamin D categories. The study did not test supplementation efficacy [58]. Pulmonary investigation should be directed by symptoms, deformity severity, and anesthesia requirements.

Preoperative optimization should translate the assessment into an individualized plan. Review and address anemia, inadequate intake, recent weight loss, uncontrolled respiratory disease, dehydration, and relevant medication exposure before elective surgery when feasible. Establish baseline bowel symptoms and nutritional needs so that postoperative change can be recognized. Agree on the anticipated fusion extent, instrumentation strategy, blood-conservation measures, analgesia, and postoperative monitoring with the surgical and anesthesia teams. Complex cases may benefit from multidisciplinary review of the imaging and the planned correction, particularly when unusual anatomy or proximity to thoracic or abdominal structures increases concern [28,40,44,59,60]. Counseling should include realistic recovery expectations and the symptoms that require reassessment. These measures represent modifiable targets, while curve severity and fixed anatomical vulnerability also inform risk but cannot simply be eliminated.

### 5.2. Intraoperative Management

Blood conservation includes correction of relevant preoperative anemia, meticulous hemostasis, appropriate positioning, and an individualized anesthetic plan [59]. A multicenter randomized trial protocol planned to enroll 150 adults undergoing spinal deformity surgery to compare high-dose TXA (50 mg/kg loading dose followed by 20 mg/kg/h) with low-dose TXA (10 mg/kg followed by 1 mg/kg/h). Planned primary outcomes included perioperative blood loss, drain output, and transfusion rates. The publication described the study design without reporting comparative efficacy or safety results [61]. In a randomized placebo-controlled trial of 111 patients, Goobie et al. reported lower mean intraoperative blood loss with tranexamic acid (TXA), 836 versus 1031 mL; six placebo-treated patients and no TXA-treated patients received allogeneic transfusion [62]. These findings support TXA for reducing blood loss and transfusion requirements. A meta-analysis of five studies involving 334 patients found that high-dose intravenous tranexamic acid reduced blood loss during AIS surgery, although between-study heterogeneity was substantial (I^2^ = 82%). No significant thromboembolic complications were reported; however, the limited sample size and inclusion of retrospective studies preclude firm conclusions regarding safety, particularly for rare adverse events [63].

Hasan et al. retrospectively compared two TXA regimens in 129 patients aged 10–21 years. Mean blood loss was 723.3 mL with a single bolus and 819.4 mL with a bolus followed by infusion (*p* = 0.065); no patient in either group received transfusion [64]. The study did not demonstrate superior efficacy of the infusion regimen. For clinical use, dosing and monitoring should follow the institutional pediatric anesthesia protocol, taking contraindications and renal function into account. These relatively small studies cannot exclude rare adverse effects.

The abdomen should be positioned to avoid unnecessary compression. Fluid replacement and transfusion should respond to physiology and measured loss. Pediatric spinal-deformity guidance emphasizes maintaining adequate spinal cord perfusion, particularly during instrumentation, correction, or a neuromonitoring change, with blood pressure targets adapted to age and operative circumstances [44]. Blood conservation should preserve neural and systemic perfusion throughout the procedure.

Other modifiable intraoperative factors include retractor placement, protection of the pleura, avoidance of unnecessary abdominal compression, and selection of a screw trajectory and length appropriate to the rotated vertebra and adjacent structures. Unexpected resistance, suspected anterior or lateral breach, unexplained bleeding, or a new ventilatory abnormality should prompt reassessment before proceeding. Navigation or other intraoperative imaging may be selected according to anatomical complexity and local expertise, but it does not replace direct assessment or guarantee prevention of vascular and visceral injury [2,19,26,28,33,34]. Communicate substantial blood loss, prolonged hypotension, suspected pleural injury, and instrumentation concerns to the postoperative team so that surveillance addresses the actual intraoperative events.

### 5.3. Postoperative Care

Multimodal analgesia can reduce opioid exposure while preserving pain control sufficient for breathing and mobilization. The regimen should be agreed upon by pediatric anesthesia and the spine team and tailored to patient factors. Antiemetic treatment, oral intake as tolerated, and mobilization when physiologically stable are central elements of many enhanced recovery pathways [18,65]. A retrospective AIS study by Fletcher et al. associated three postoperative dexamethasone doses with reduced opioid consumption: 39.6% for total morphine equivalents and 29.5% after weight normalization. Wound complications did not differ significantly between groups, but the study was not powered to establish the absence of uncommon harm [66].

Postoperative bowel plans should distinguish constipation from ileus and obstruction. Routine documentation of intake, vomiting, abdominal distension, flatus, and bowel movements can support recognition of a changing course. No specific laxative, peripheral opioid antagonist, or chewing-gum regimen has sufficiently consistent AIS evidence to be described as universally effective [36,39,46].

Postoperative care should target recovery barriers that remain modifiable: excess sedation or opioid exposure, inadequate analgesia for breathing and walking, persistent nausea, insufficient intake, dehydration, electrolyte disturbance, and avoidable immobility. Reassess respiratory status and oxygen requirement alongside bowel function rather than treating these as unrelated recovery milestones. Record the trend in symptoms and the interventions required; earlier mobilization or discharge alone does not demonstrate that a specific complication has been prevented [18,36,65,67]. Persistent inability to sustain intake should trigger renewed nutritional and diagnostic assessment. Before discharge, provide a clear route for urgent advice and instructions on bilious or persistent vomiting, increasing distension, dyspnea, syncope, and unexplained worsening pain. Continued review of weight and intake is particularly relevant when SMAS is a concern [37,40,68].

## 6. Enhanced Recovery and Quality Improvement

The most consistent finding across AIS enhanced recovery studies is an association with shorter hospitalization [69]. In the prospective two-center study by Fletcher et al., 203 patients on an ERAS pathway had a mean stay of 2.2 days compared with 4.8 days among 73 patients on a traditional pathway. Pain at discharge and measured recovery at follow-up were similar. The groups differed in curve magnitude, fusion extent, osteotomy use, operative duration, and blood loss, limiting attribution of all differences to the pathway alone [18].

Other institutional studies report shorter stays after pathway implementation, with varying effects on analgesia and recovery milestones [65,70,71,72]. Sundar et al. reported lower recorded POI and readmission rates after a standardized care pathway [67]. That result supports an association with the entire care bundle, not proof that any one component caused the reduction. In the quality-improvement study by Hengartner et al., both operative practice and postoperative care changed, including the number of levels fused and use of drains [60].

Table 3 summarizes selected comparative enhanced recovery cohorts and a separately identified quality-improvement initiative. Differences in design, case mix, outcome definitions, and observation periods limit comparison. Shorter length of stay should be interpreted alongside readiness for discharge, emergency visits, readmissions, pain, and patient-reported recovery.

## 7. Discussion and Practical Application

This review highlights the importance of interpreting thoracic and abdominal complications after PSF for AIS according to their clinical significance and method of detection. Reported frequencies vary because studies measure different outcomes, including imaging abnormalities, symptoms, coded diagnoses, and events requiring intervention. Bartley et al. evaluated major complications, whereas Keil et al. captured a broader spectrum of postoperative morbidity [3,5]. These findings describe complementary aspects of recovery. For counseling and comparison between institutions, complication reporting should therefore specify severity, treatment requirements, and observation periods. A single overall complication percentage provides limited guidance unless these underlying definitions are explicit. For this review, clinical significance is described by four categories of consequence. These categories are a practical reporting framework, not a validated severity scale, and should not replace a reported modified Clavien–Dindo–Sink grade when that system was used [5].

(1) Imaging finding only: an abnormality without attributable symptoms, physiological impairment, or additional treatment, such as a small CT-detected pleural effusion in an otherwise stable adolescent [9]. Its detection should be reported separately from symptomatic morbidity.

(2) Symptomatic but self-limited: transient symptoms that resolve with observation or routine supportive care and do not cause organ dysfunction or require an invasive procedure. Examples may include brief constipation or nausea. Duration, effects on oral intake and activity, and any delay in recovery should still be recorded, because self-limited symptoms can be important to patients.

(3) Requiring intervention: an event that leads to additional complication-directed treatment beyond routine recovery care, such as nasogastric decompression, supplemental nutritional support, pleural drainage, endoscopy, embolization, or reoperation. The exact intervention and its indication should be stated; this broad category includes treatments of very different burdens.

(4) Life-threatening: an event with actual or imminent organ failure, severe respiratory compromise, hemodynamic instability, shock, bowel ischemia or perforation, or another need for emergency resuscitation or organ support. Examples include tension pneumothorax, uncontrolled hemothorax, and abdominal compartment syndrome [12,43]. Such events should be reported separately even when they also require an intervention.

These categories describe the observed course rather than permanently assigning each diagnosis to one level. An effusion may remain an imaging finding or progress to respiratory compromise; ileus may resolve with supportive care or require decompression and nutritional treatment. For comparative reporting, record the highest consequence reached together with symptoms, treatment, onset, recovery, and residual impairment. In this framework, clinically significant morbidity includes categories 2 through 4, with intervention-requiring and life-threatening events reported separately; category 1 represents a surveillance finding. This definition is an explicit convention for interpretation in this review, rather than a claim that the included studies used a common definition.

**Figure 3:**
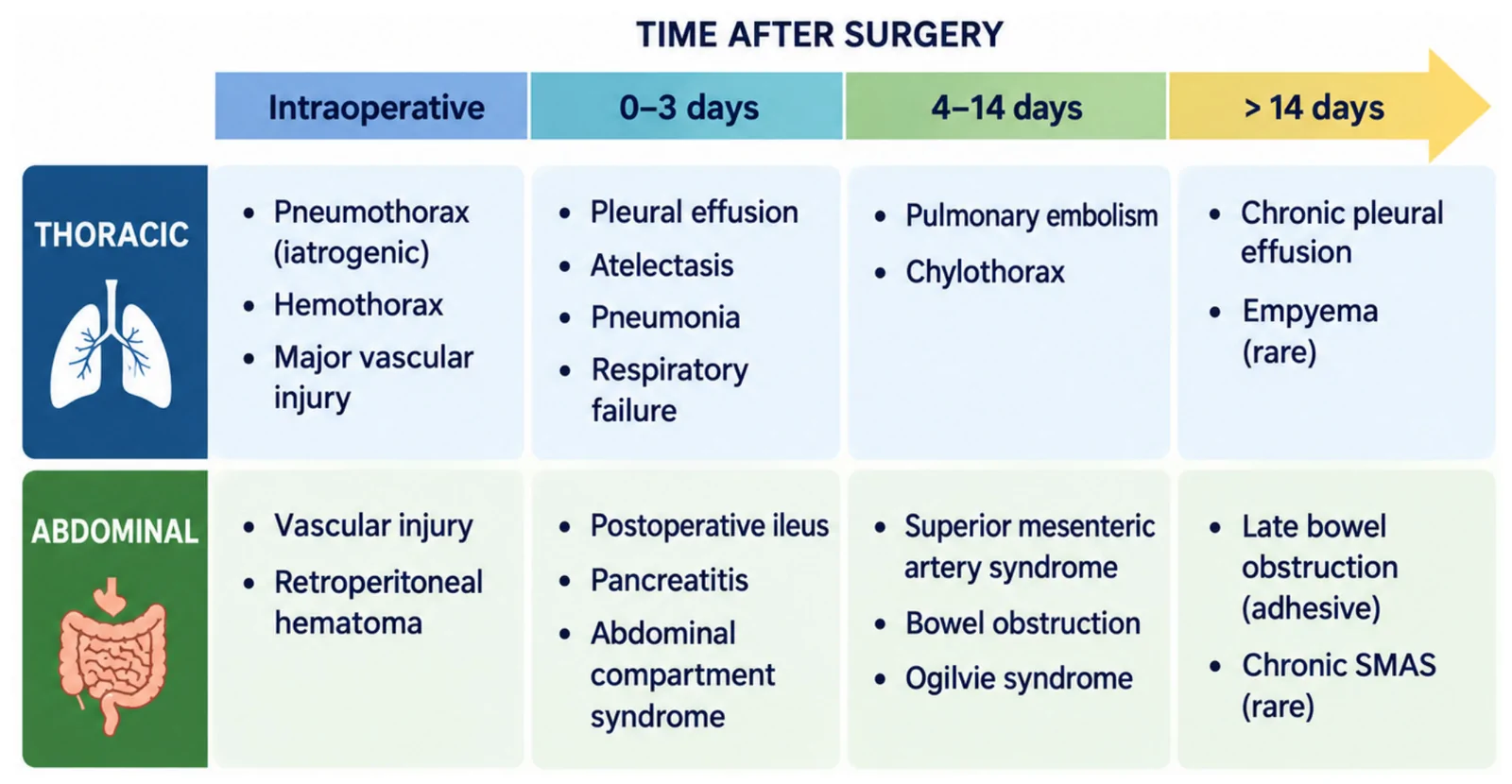
Timeline of onset of selected thoracic and abdominal complications after PSF. Clinical vigilance and appropriate monitoring should be tailored to the expected timing of complications. The figure was prepared using code developed with assistance from ChatGPT (OpenAI, USA; https://chatgpt.com/; accessed on 17 September 2026). The authors take responsibility for the figure’s content and accuracy.

Pleural effusion illustrates this distinction particularly clearly. Studies using CT approximately one week after surgery identified pleural fluid in most patients [7,9]. In the study by Ozaki et al., all detected effusions were asymptomatic, required no treatment, and were not associated with significant differences in pulmonary function or lung volume at two years [9]. These observations support conservative interpretation of incidental fluid in clinically stable patients. They should not be generalized to symptomatic collections or patients undergoing additional thoracoplasty, for whom pleural effusion was associated with longer oxygen requirements and intensive care stays [10]. Differences in ascertainment also preclude direct comparison of these percentages. Figure 3 shows the timeline of onset of selected thoracic and abdominal complications after PSF.

The practical consequence is that postoperative imaging should address a clinical question. Esposito et al. found that pneumothorax requiring intervention was uncommon, questioning the value of routine chest radiographs after uncomplicated AIS fusion [11]. However, such findings do not reduce the importance of prompt investigation when oxygen requirements increase or respiratory deterioration occurs. General thoracic trauma guidance supports immediate treatment of suspected tension pneumothorax with physiological compromise, without delaying intervention for imaging [12]. Thus, selective imaging and vigilance are compatible approaches: avoiding unnecessary screening does not imply a higher threshold for investigating an abnormal postoperative course.

Abdominal symptoms require similar discrimination. The POI frequency reported by Zhang et al. depended on a definition requiring at least two gastrointestinal features at 72 h [35]. This research definition should not become a clinical waiting period when bilious vomiting, progressive distension, or systemic deterioration suggests obstruction or another complication. Associations between POI, increased spinal height, and anterior lumbar screw breach are potentially informative, but remain observational [35]. They do not establish a safe correction threshold or prove that deliberately limiting correction prevents ileus. Clinical assessment should integrate the trajectory of symptoms with examination, medication exposure, and appropriate investigation.

Nutritional vulnerability deserves attention because it may interact with the anatomical changes produced by deformity correction. Reports of SMAS associate low body mass and postoperative weight loss with susceptibility to duodenal compression [37,40,41]. However, these studies do not establish a universally effective weight target or preventive nutritional protocol. Assessment should therefore consider growth, recent weight change, and the ability to maintain intake, rather than relying on a single absolute BMI threshold. The widely variable timing of reported SMAS presentations also supports continued attention after discharge [37]. Persistent vomiting should prompt assessment for obstruction even when initially attributed to medication or routine gastrointestinal recovery.

**Figure 4:**
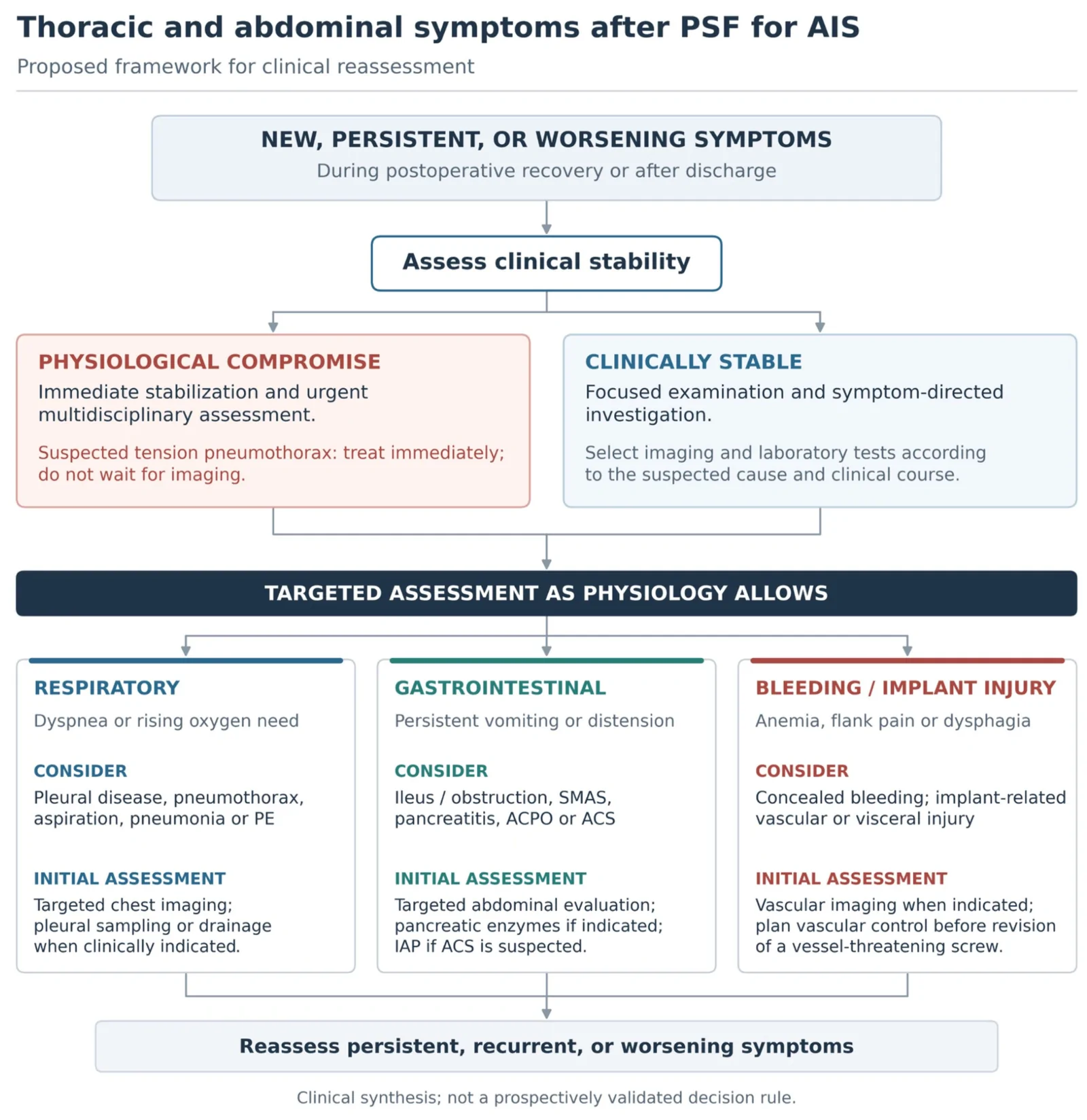
Proposed framework for assessing thoracic and abdominal symptoms after posterior spinal fusion for adolescent idiopathic scoliosis. This figure synthesizes the evidence and clinical implications discussed in the review and is not a prospectively validated decision rule. Reference numbers correspond to the manuscript bibliography. Abbreviations: ACPO, acute colonic pseudo-obstruction; ACS, abdominal compartment syndrome; AIS, adolescent idiopathic scoliosis; IAP, intra-abdominal pressure; PE, pulmonary embolism; PSF, posterior spinal fusion; SMAS, superior mesenteric artery syndrome [11,12,13,14,15,16,21,22,30,31,32,35,37,43,53,54,55]. The figure was prepared using code developed with assistance from ChatGPT (OpenAI, USA; https://chatgpt.com/; accessed on 17 September 2026). The authors take responsibility for the figure’s content and accuracy.

Postoperative pancreatitis provides another example of diagnostic heterogeneity. Administrative data, historical symptom-triggered enzyme testing, and prospective clinical assessment have yielded markedly different frequencies [42,50,51]. These estimates cannot define a single contemporary risk after posterior-only AIS surgery. Furthermore, El Bouyousfi et al. reported abdominal pain in only half of their postoperative cases, indicating that delayed gastrointestinal recovery may be the presenting concern [52]. Applying pediatric diagnostic criteria requiring two of three clinical, biochemical, and imaging features helps avoid equating isolated enzyme elevation with pancreatitis [53]. Early recognition should lead to reassessment of hydration, nutrition, organ function, and alternative explanations for persistent symptoms. Figure 4 summarizes a proposed approach to reassessment of new, persistent, or worsening thoracic and abdominal symptoms after PSF for AIS. Clinical stability determines the urgency of initial management, while the presenting symptom pattern guides subsequent investigation. The diagnostic groups overlap and do not constitute an exhaustive differential diagnosis. Investigations and treatment should be adapted to patient age, physiology, operative history, and available expertise.

Mechanical duodenal compression in SMAS should also be distinguished from vascular compression with visceral ischemia. The two cases described by Včelák et al. suggest that acute abdominal deterioration after correction can occasionally involve a vascular mechanism requiring reconsideration of the spinal correction [38]. Their successful revisions illustrate a possible management response in selected circumstances, rather than a general treatment strategy for postoperative pancreatitis. Likewise, ACPO and ACS require separate diagnostic frameworks: colonic dilatation without mechanical obstruction differs from elevated intra-abdominal pressure with attributable organ dysfunction [43,54,55]. Table 4 summarizes warning findings while preserving these distinctions and the need for individualized multidisciplinary assessment. The proposed approach synthesizes available evidence and clinical reasoning; it has not been prospectively validated as a diagnostic algorithm or risk prediction model.

Instrumentation safety remains central, but radiographic screw accuracy and clinical injury are distinct outcomes. Screw assessments can identify cortical breaches without corresponding neurological or vascular complications [26,27]. Conversely, reports of delayed hemothorax and esophageal perforation show that implant-related injury may become apparent years after surgery [21,30]. New thoracic symptoms or dysphagia therefore warrant consideration of the existing instrumentation. When vascular involvement is suspected, imaging and coordination with vascular specialists should precede hardware removal; contextual reports illustrate approaches to securing vascular control [31,32]. These observations support careful investigation of symptomatic patients without establishing a routine imaging schedule for every asymptomatic patient. Evidence concerning navigation requires similarly cautious interpretation. Zhao et al. associated O-arm use with lower pleural fluid volume and fewer screw perforations in a small matched comparison [8]. Larger database studies have reported conflicting associations: Chan et al. found fewer neurological injuries and transfusions, whereas Singh et al. reported more recorded complications, particularly transfusions [2,34]. Differences in databases, coding, patient selection, and operative complexity may contribute to these discrepancies. Neither result establishes that navigation independently prevents rare visceral or vascular injuries. Future comparisons should distinguish screw placement accuracy from patient outcomes and account for the clinical reasons underlying navigation use.

Complex preoperative planning should connect the individual anatomy to the intended correction and to a plan for managing foreseeable injury. Review of vertebral rotation, pedicle dimensions, adjacent vessels and viscera, anticipated screw length, and the proposed fusion levels can identify where additional imaging, an alternative anchor, navigation, or specialist input may be useful [28,33,60]. Three-dimensional reconstruction or physical modeling may assist selected complex cases when it answers a specific anatomical question; the available evidence does not justify routine additional CT solely to create a model in every adolescent.

As an explicitly contextual example, Solyom et al. described a six-stage workflow incorporating image acquisition, virtual and physical modeling, preoperative simulation, surgery, and imaging validation in a single patient with a complex acetabular fracture [73]. That report illustrates a planning method and the review of implant trajectories, but it concerns pelvic trauma rather than PSF for AIS. Its single-case design provides no comparative complication-reduction rate and cannot establish the effectiveness of this method in scoliosis surgery. A descriptive report presented a structured preoperative planning method for AIS, supported by free semiautomated digital software and illustrated in three cases treated with posterior spinal fusion. Combining traditional planning principles with digital measurements, the approach offers an accessible, low-cost framework for fusion-level selection, operative preparation, and surgical training [74].

More directly applicable evidence comes from the retrospective AIS study by Hengartner et al., which included 44 patients before and 52 after a quality-improvement initiative incorporating monthly multidisciplinary conferences. The proportion of patients experiencing any postoperative complication was 72.7% before implementation and 34.6% afterward (*p* < 0.001) [60]. This quantified association concerns a broad complication outcome and a program that also changed fusion extent, drain use, and recovery care. It cannot isolate the contribution of preoperative planning or establish the reduction in rare thoracic or abdominal events. Planning should therefore be presented as part of coordinated perioperative risk management, while a planning-specific percentage reduction remains unproven.

Prevention should focus on outcomes for which supporting evidence is identifiable. The randomized trial by Goobie et al. supports TXA for reducing blood loss and transfusion requirements in AIS surgery [62]. A meta-analysis of 10 studies involving 741 patients with AIS found that high-dose tranexamic acid reduced intraoperative, postoperative, and total blood loss, as well as transfusion requirements (RR, 0.28; 95% CI, 0.17–0.45). In a randomized study of 90 patients undergoing PSF for AIS, adding 2 g of topical TXA through retrograde drain instillation with 2-h clamping to intravenous TXA reduced postoperative drainage volume (372.0 versus 545.2 mL; *p* < 0.001), drain duration, and hospital stay compared with intravenous TXA alone. Transfusion and complication rates were comparable between groups [75]. Reported thromboembolic event rates were comparable between groups, although these findings do not exclude rare adverse effects [76]. However, blood conservation must be considered alongside tissue perfusion. Pediatric spinal deformity recommendations emphasize maintaining spinal cord perfusion throughout correction and postoperative care [44]. The association between lower intraoperative arterial pressure and pancreatitis in the prospective scoliosis cohort of Feng et al. provides additional reason to examine hemodynamic factors, although it does not establish causality or a universal blood pressure threshold for preventing abdominal complications [42]. Thromboprophylaxis similarly requires balancing a low population event frequency against individual risk. Rudic et al. identified associations between VTE and thrombophilia, obesity, longer fusion, and older adolescent age [22]. The ASH/ISTH guideline conditionally suggests against routine anticoagulant prophylaxis for pediatric noncardiac surgery, based on very low-certainty evidence, while recognizing the need for individual assessment [25]. This broad recommendation should not be interpreted as excluding prophylaxis in every higher-risk adolescent. Equally, adult protocols cannot be adopted automatically. Clinical decisions should consider thrombotic risk, mobility, and bleeding consequences in consultation with the appropriate pediatric team.

Enhanced recovery pathways offer consistent evidence of shorter hospitalization, but their effects require careful attribution. A systematic review of 10 studies comparing 1040 patients managed with ERAS protocols and 959 receiving conventional care after posterior surgery for AIS found that ERAS was associated with shorter hospitalization (*p* < 0.00001). No statistically significant differences were detected in complication or readmission rates. However, the predominantly retrospective, nonrandomized evidence limits causal inference and does not establish equivalence in safety outcomes [77]. A meta-analysis of 30 studies found that ERAS pathways in AIS surgery were associated with shorter hospitalization (mean reduction, 1.7 days), earlier ambulation (37.4 h), and fewer overall complications compared with traditional care (4% versus 8%; *p* = 0.0074). Wound-related complications and 30-day readmission rates were comparable between groups [47]. In the prospective comparison by Fletcher et al., groups differed in curve magnitude, osteotomy use, fusion extent, operative duration, and blood loss [18]. These differences complicate estimation of an independent pathway effect. Sundar et al. reported fewer ileus episodes and readmissions after a standardized pathway, whereas Hengartner et al. evaluated an initiative incorporating changes in both operative practice and postoperative care [60,67]. Such findings support coordinated care while leaving uncertainty about which components produce benefit and whether they prevent individual rare complications.

Gastrointestinal and analgesic interventions illustrate the limitations of component-specific evidence. A systematic review found limited support for particular bowel regimens; methylnaltrexone showed favorable associations in a retrospective matched study, while randomized trials showed no significant improvements in abdominal pain, nausea, or hospitalization length with chewing gum [36,39,46]. Postoperative dexamethasone was associated with lower opioid consumption, but the study could not reliably exclude uncommon adverse effects [66]. Moreover, cumulative opioid exposure during hospitalization is influenced by hospitalization length. Future studies should compare doses over standardized postoperative intervals, alongside pain, mobilization, bowel recovery, and prescribing after discharge, rather than relying exclusively on total inpatient consumption.

Recovery assessment should extend beyond discharge. Harris et al. identified emergency department attendance in 9.7% of AIS fusion patients within six months, commonly for pain and gastrointestinal concerns [68]. Although this study did not test discharge education, its findings support evaluating communication, access to advice, and symptom recognition as components of care. Families should receive clear instructions concerning persistent vomiting, respiratory symptoms, and progressive abdominal complaints. Follow-up should assess oral intake, weight trajectory, functional recovery, and unplanned healthcare use. Earlier discharge is most informative when interpreted alongside these outcomes and consistent discharge criteria.

This review is limited by its narrative selection process, heterogeneous definitions, retrospective evidence, and dependence on case reports for rare events. Contextual studies and general guidance extend clinical coverage but reduce certainty for posterior-only AIS. Future research should prioritize multicenter event adjudication, consistent denominators, and separation of radiographic findings from symptomatic disease. Studies should report intervention requirements and timing, identify potentially overlapping registry populations, and include outcomes after discharge. These improvements would make complication estimates more useful for counseling and allow prevention strategies to be evaluated against clinically meaningful outcomes.

The absence of reconstructable retrieval and screening counts is an additional methodological limitation. Reporting the final bibliography and a descriptive selection schematic improves transparency but cannot establish search completeness, quantify excluded evidence, or remove selection bias. Likewise, the consequence categories proposed here clarify interpretation but have not been validated prospectively and do not retrospectively standardize the definitions used by the original studies.

## 8. Future Research

Future studies should use a core set of definitions separating imaging findings, clinical diagnoses, treatments, severity, and time of onset. AIS cohorts should be identified independently of other deformities, with surgical approach, thoracoplasty, age range, and instrumentation extent reported. For rare complications, multicenter registries with adjudicated events are more informative than isolated institutional percentages.

ERAS research should evaluate the whole pathway and its implementation while accounting for operative complexity and changes in discharge practice [69]. Trials or carefully designed comparative studies are needed for bowel regimens and analgesic adjuncts. Navigation studies should report radiation exposure, operative time, screw-level accuracy, and patient-level outcomes separately. A reduction in radiographic breach should not be assumed to produce a measurable reduction in rare vascular injuries without appropriate clinical data.

Longer follow-up is necessary to capture delayed implant-related injury and events occurring after discharge. Patient-reported recovery, nutritional function, unplanned care, and family burden should complement length of stay. Risk models should be validated before their factors are combined into a clinical score or used to mandate additional testing.

## 9. Conclusions

Thoracic and abdominal complications after PSF for AIS encompass frequent transient findings and uncommon but serious disorders. Frequency estimates are meaningful only when the population, denominator, diagnostic method, and follow-up are stated. CT-detected pleural fluid should be distinguished from symptomatic pleural disease, and postoperative constipation from ileus and obstruction.

Blood conservation, appropriate perfusion, accurate instrumentation, individualized nutrition, multimodal analgesia, and coordinated recovery pathways support perioperative care. Evidence is strongest for selected outcomes such as blood loss and length of stay; prevention of individual rare complications remains less certain. Prompt recognition of an abnormal recovery trajectory and multidisciplinary treatment are essential when respiratory compromise, obstruction, pancreatitis, concealed bleeding, or vascular injury is suspected.

In practice, clinicians should use the preoperative visit to identify and address nutritional vulnerability, recent weight loss, anemia, and relevant respiratory disease, and should agree on instrumentation, perfusion, and recovery plans before surgery. During the operation, protect the pleura and adjacent structures, maintain appropriate perfusion, and reassess suspected screw breach or unexplained bleeding. After surgery, combine effective opioid-sparing analgesia with symptom and intake monitoring, correction of fluid and electrolyte deficits, and mobilization when stable. Observe small asymptomatic pleural collections according to the clinical course, while promptly investigating increasing oxygen need or respiratory symptoms. Persistent or bilious vomiting, progressive distension, focal abdominal pain, unexplained anemia, and hemodynamic deterioration should trigger assessment for a specific complication rather than be accepted as routine recovery. Discharge instructions and follow-up should address these warning symptoms, oral intake, and weight change. Finally, record whether an event was an imaging finding, self-limited symptomatic morbidity, intervention-requiring, or life-threatening so that counseling and quality assessment reflect consequences for the patient.

## Figures and Tables

**Table 1:** Selected thoracic outcomes and their clinical interpretation.

Outcome and Source	Population and Ascertainment	Reported Result	Interpretation
Pleural effusion, Hayashi et al. [7]	76 AIS patients; CT at one week	54/76 (71.0%); bilateral in 36/76 (47.4%)	Imaging prevalence; not a drainage or respiratory-failure rate
Pleural effusion, Ozaki et al. [9]	114 AIS patients; CT at one week	87/114 (76.3%); none symptomatic or treated	Subclinical finding in this cohort
Effusion after thoracoplasty, Amanullah et al. [10]	45 AIS patients with concomitant thoracoplasty	Effusion 16/45 (35.6%); chest tubes 3/45 (6.7%)	Selected surgical subgroup
Pneumothorax, Esposito et al. [11]	National database and institutional PSF cohorts	30/9036 (0.3%) and 8/244 (3.3%); intervention 4/9036 and 0/244	Ascertainment differs; not pleural-effusion data
Pneumonia, Basques et al. [16]	733 AIS PSF patients; pediatric surgical database; 30-day follow-up	1/733 (0.1%)	Recorded clinical event
VTE, Rudic et al. [22]	11,775 AIS instrumented fusions; 90-day claims	38/11,775 (0.323%)	Combined VTE; not PE alone
Chylothorax, hemothorax, vascular injury [13,14,21]	Predominantly case reports	No reliable AIS-specific incidence	Establish possibility and presentation, not absolute risk

AIS, adolescent idiopathic scoliosis; CT, computed tomography; PE, pulmonary embolism; PSF, posterior spinal fusion; VTE, venous thromboembolism. Percentages are study-specific and should not be pooled across these rows.

**Table 2:** Abdominal outcomes and limits of available frequency estimates.

Outcome and Source	Population or Evidence	Reported Result	Interpretation
POI, Zhang et al. [35]	318 patients with AIS, ages 10–20 years; ≥2 specified gastrointestinal features at 72 h	42/318 (13.2%)	Apply the study definition; not interchangeable with constipation
SMAS, Kwan et al. [4]	1057 AIS PSF patients, ages 10–30 years	2/1057 (0.19%)	Single-center, mixed adolescent and young-adult age range
SMAS, Pappalardo et al. [37]	29 publications, 61 reported patients	Case-based synthesis	No denominator for a pooled PSF incidence
Pancreatitis, Laplaza et al. [51]	80 AIS patients operated on in 1995–1997; symptom-triggered enzyme testing in 12	7/80 (approximately 9%) described as pancreatitis	Historical diagnostic criteria and selective testing; limited comparison with current pediatric diagnoses
Pancreatitis, De la Garza Ramos et al. [50]	36,335 AIS patients aged 10–18 years; NIS 2002–2011; mixed surgical approaches; index hospitalization	≤0.2%	Administrative estimate; not specific to posterior-only surgery
Pancreatitis, Feng et al. [42]	176 patients undergoing posterior scoliosis correction; prospective cohort; clinical and laboratory diagnosis	13/176 (7.4%)	Contextual scoliosis-surgery estimate; not treated as an AIS-specific incidence
ACPO [54]	Case report involving Chiari malformation and syringomyelia	No AIS denominator	Contextual evidence only
Retroperitoneal hematoma and ACS [26,43]	Anatomical risk evidence and general consensus	No reliable AIS-specific estimate	Retain in the differential; do not assign an unsupported percentage

ACPO, acute colonic pseudo-obstruction; ACS, abdominal compartment syndrome; AIS, adolescent idiopathic scoliosis; NIS, Nationwide Inpatient Sample; POI, postoperative ileus; PSF, posterior spinal fusion; SMAS, superior mesenteric artery syndrome.

**Table 3:** Selected comparative AIS studies of enhanced recovery and quality improvement.

Study and Design	Pathway and Comparator Groups	Mean Stay, Days	Other Relevant Findings and Limitations
Fletcher et al., 2021 [18]; prospective two-center comparison	ERAS 203; traditional 73	2.2 vs. 4.8	Similar discharge pain and follow-up recovery; baseline and operative differences
Spisak et al., 2023 [70]; retrospective comparison	ERAS 20; traditional 22	3.3 vs. 5.0	Opioid use 1.4 vs. 2.4 morphine equivalents/kg; small selected cohort
Jeandel et al., 2023 [71]; comparison with historical controls	ERAS 30; control 30	5.0 vs. 6.5	Lower early pain; one postoperative complication in each group
Temby et al., 2021 [72]; pre/post implementation	Enhanced recovery 61; conventional 32	3.5 vs. 6.3	Earlier milestones; no detectable difference in complications or readmissions
Rao et al., 2021 [65]; retrospective before/after study	Post-pathway 39; pre-pathway 78	3.8 vs. 4.6	Lower early opioid use; no change in readmission rate
Sundar et al., 2022 [67]; retrospective care-path study	Post-pathway 219; pre-pathway 117	3.36 vs. 4.14	Recorded POI 1.9% vs. 8.5%; 60-day readmissions 0.48% vs. 6.0%
Hengartner et al., 2024 [60]; retrospective quality-improvement study	Post-initiative 52; pre-initiative 44	3.2 vs. 4.5	Any complication 34.6% vs. 72.7%; operative differences and broad outcome definition

Values are presented as pathway/post-implementation versus comparator/pre-implementation. AIS, adolescent idiopathic scoliosis; ERAS, enhanced recovery after surgery; POI, postoperative ileus. This is a descriptive comparison, not a meta-analysis. Complication definitions and observation periods differ.

**Table 4:** Findings that should prompt reassessment after PSF.

Clinical Finding	Important Possibilities	Initial Response and Escalation
Acute respiratory or hemodynamic deterioration	Tension pneumothorax, major bleeding, aspiration, PE	Immediate physiological assessment and stabilization; do not delay treatment of suspected tension physiology for imaging [12,22]
Progressive pleural fluid or rising oxygen requirement	Symptomatic effusion, chyle, hemothorax, infection	Targeted imaging; fluid analysis or drainage according to the suspected cause and clinical effect [9,13,15,21]
Bilious vomiting or failure to sustain intake	SMAS, mechanical obstruction, severe ileus	Reassess hydration and abdomen; decompression when indicated; targeted imaging and pediatric surgical review [35,37]
Persistent epigastric pain or unexplained digestive delay	Pancreatitis and competing abdominal disease	Check diagnostic criteria; obtain enzymes and selective imaging; monitor fluid and nutritional needs [53]
Progressive distension with systemic deterioration	ACPO, ischemia, perforation, ACS	Urgent surgical assessment; exclude obstruction or ischemia and measure pressure when ACS is suspected [43,55]
Unexplained anemia, flank pain, or vascular contact by a screw	Concealed bleeding or implant-related vessel injury	Assess circulation and bleeding; vascular imaging when stable; plan vascular control before hardware revision [31,32]

ACPO, acute colonic pseudo-obstruction; ACS, abdominal compartment syndrome; PE, pulmonary embolism; PSF, posterior spinal fusion; SMAS, superior mesenteric artery syndrome. The actions require adaptation to age, physiology, and available expertise.

## Data Availability

Data is contained within the article.
